# Novel utilization of 3D technology and the hybrid operating theatre: Peri-operative assessment of posterior sterno-clavicular dislocation using cone beam CT

**DOI:** 10.1002/jmrs.15

**Published:** 2013-06-03

**Authors:** James A Crowhurst, Douglas Campbell, Mark Whitby, Pavthrun Pathmanathan

**Affiliations:** The Prince Charles HospitalRode Road, Chermside, Queensland, Australia

**Keywords:** Cone beam CT, hybrid theatre, peri-operative, sterno-clavicular dislocation

## Abstract

A patient with a medial and posterior dislocation of the right sterno-clavicular (SC) joint and displacement of the trachea and brachiocephalic artery by the medial head of the clavicle underwent general anaesthetic in the operating theatre for an open reduction procedure. The surgeon initially attempted a closed reduction, but this required imaging to check SC alignment. The patient was transferred to an adjacent hybrid operating theatre for imaging. Cone beam computed tomography (CBCT) was performed, which successfully demonstrated a significant reduction in the dislocation of the SC joint. The trachea and brachiocephalic artery were no longer compressed or displaced. This case study demonstrates an alternative to the patient being transferred to the medical imaging department for multi-slice CT. It also describes a novel use of the hybrid operating theatre and its CBCT capabilities.

## Introduction

Traumatic posterior sterno-clavicular (SC) joint dislocation is rare, accounting for less than 1% of all dislocations reported. It is usually brought about by significant blunt trauma to the medial end of the clavicle and was first described in 1824. The condition has been associated with multiple complications, including respiratory compromise, brachial plexopathy, pneumothorax, dysphagia, vascular injury, and even death.^1^ Important structures are found in close proximity to the SC joint; those traversing the superior mediastinum and thoracic outlet are in particular danger when the medial end of the clavicle is displaced posteriorly^2^; consequently, the condition should be managed in a timely manner. Plain film radiography has difficulty assessing posterior SC joint dislocation, but views such as the “Rockwood” view with a 50 degree cranial angulation can be helpful in the initial assessment.^3^ Multi-slice computed tomography (MSCT) is optimal in diagnosing this condition, as multi-planar reconstructions can be performed and any vascular compromise can be demonstrated through intravenous contrast media.^3^ Cone beam computed tomography (CBCT) has emerged in the last decade, providing MSCT-like images in the angiography suite, aiding complex percutaneous interventions.^4^ The hybrid operating theatre (an angiography suite in a fully sterile operating theatre) is a new concept, designed predominantly for cardiovascular and endovascular procedures; however, this case study demonstrates the novel use of a hybrid theatre for the imaging of posterior SC joint dislocation.

## Case Report

**Figure 1:**
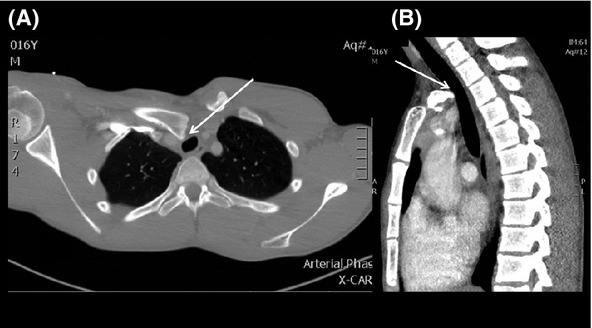
Multi-slice computed tomography demonstrating posterior dislocation of the sterno-clavicular joint and abutment and displacement of the trachea. These pre-operative images, (A) axial and (B) sagittal, demonstrate how the medial end of the clavicle is dislocated and is abutting and compressing the trachea (arrow).

An 18-year-old male suffered a medial and posterior dislocation to the right SC joint during a rugby tackle. This was confirmed by MSCT performed at a regional hospital. The MSCT also demonstrated that the medial head of the clavicle had abutted and displaced the trachea and brachiocephalic artery (Fig. 1). The patient was then transferred to a tertiary cardiothoracic hospital.

**Figure 2:**
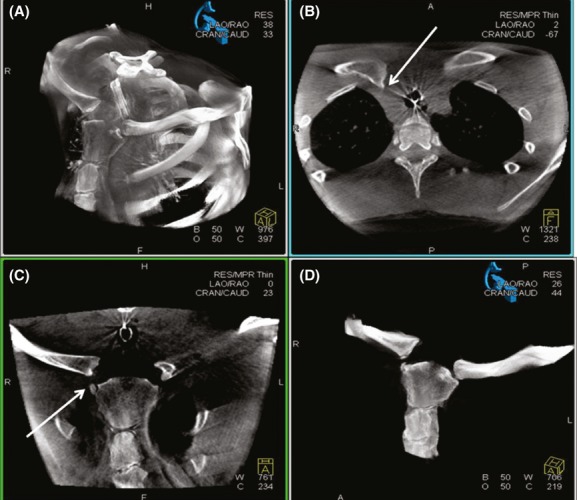
Cone beam computed tomography (CT) performed in the hybrid theatre demonstrating adequate reduction in the sterno-clavicular joint. The above images demonstrate the anatomy from the cone beam CT scan immediately after closed reduction in the posterior sterno-clavicular dislocation. (A) An overall volume-rendered 3D image reconstruction of the anatomy. (B) An axial plane reconstruction and how the medial end of the clavicle is no longer compressing the trachea (arrow). (C) Coronal reconstruction demonstrating a small avulsion fracture of the lateral aspect of the sternum (arrow). (D) An oblique 3D reconstruction demonstrating improved alignment of the clavicle in relation to the sternum.

In the operating theatre, a general anaesthetic was administered for a planned open reduction in the SC joint. The orthopaedic surgeon initially attempted a closed reduction of the clavicle into a more normal position, but confirmation of a satisfactory result was required through imaging. Discussions were held around transferring the patient to the medical imaging department for a CT scan of the chest; however, after consulting with the senior radiographer of the theatre area and the radiology medical director it was deemed appropriate to transfer the patient to an adjacent hybrid operating theatre for three-dimensional (3D) imaging. The hybrid theatre is equipped with a Siemens *Artis Zee* dTA (Siemens Healthcare, Erlangen, Germany) cardiovascular imaging suite with fixed ceiling-mounted C-arm with CBCT and 3D capabilities. A *DynaCT* was performed, with an 8-sec rotation time through 200 degrees, acquiring 400 images. These images were transferred to the workstation, where axial, sagittal, and coronal reformats and volume-rendered 3D images were created (Fig. 2). These images demonstrated a significant reduction in the dislocation of the SC joint and normal placement of the trachea and brachiocephalic artery. The patient was subsequently taken to theatre recovery and then to the ward shortly after. The patient was discharged the next day, pain free, with the affected arm in a sling and instructions of limited activity. On follow-up in the clinic 2 weeks later, the clavicle was still reduced and the patient had full range of movement. Within 3 months the patient was sufficiently well to ride motorbikes.

## Discussion

Owing to the rarity of this injury, there is a relative lack of familiarity with the diagnosis, surgical anatomy, and treatment options. Closed reduction techniques involve the patient lying supine over a bolster placed between the scapulae and manipulating the ipsilateral arm.^1^ Closed reduction in a posterior dislocation of the SC joint is not often achievable and an open reduction strategy is therefore favoured.^5^ In this case study, the surgeon attempted a closed reduction in the clavicle and if unsuccessful, moved to an open procedure. Consequently the surgeon had to be sure that a complete reduction had been achieved with the closed method. Although plain film radiographs could have been performed, CT is the imaging technique of choice as it can demonstrate the anatomy in multiple planes.^6^

CBCT has been given many names: C-arm CT, volume CT, cone beam volume CT, angiographic CT, rotational angiography, and flat panel CT.^4^ Its emergence dates back as far as 1984, first used with a C-arm and image intensifier,^7^ but has taken off in routine practice in the last decade with the emergence of flat panel detectors that allow for much greater contrast and spatial resolution and are now aided with greater computing power.^8^

CBCT has been shown to be useful in a variety of settings, usually guiding vascular intervention and also some orthopaedic trauma.^9^–^12^ The technique also has a role in radiotherapy planning^13^ and has been used extensively in orthodontic surgical planning for many years.^14^,^15^

The CBCT produces a cone-shaped x-ray beam that is transmitted through the patient and onto the detector, much like any standard digital radiograph. However, in CBCT, the C-arm (and therefore the x-ray tube and detector) rotate around the patient while acquiring a series of images, which produces a data set that can be manipulated into multi-planar or 3D reconstructions. The CBCT has distinct advantages in that the data are collected in one rotation, and the C-arm can be used for intervention, as it traditionally would, without moving the patient. The MSCT technique differs somewhat in that the patient is scanned in a helical fashion, with the x-ray tube and detector rotating at speeds in the order of 0.4 seconds, with multiple rows of detectors and a collimated “fan”-shaped beam of x-rays. The patient is moved on the table through the beam as data are collected. The faster rotation times mean greater temporal resolution and faster scan times for a given body area, even though the detector size is smaller.^4^

Radiation doses of these two CT techniques have been investigated on a number of occasions. Smyth et al.^16^ investigated the technique using phantoms and found the surface dose to the patient using CBCT to be significantly higher than that of MSCT, but with inferior contrast resolution. They concluded that image quality of CBCT was poorer and dose higher, although its availability in the interventional setting was a distinct advantage. Subjectively, the image quality for the CBCT in this case study was also inferior to that of the MSCT, with lower contrast resolution and more streak artefact, but the images acquired were adequate for the purposes required. To answer the question of radiation dose for the patient in this case study, dose calculations were performed by a medical physicist. Calculations were performed for both the pre-operative MSCT and the intra-operative CBCT. The effective dose of the MSCT scan performed at the regional centre was calculated to be 2.3 mSv for one acquisition and 2.9 mSv for a second, totalling 5.3 mSv. In comparison, the intra-operative CBCT effective dose was calculated to be 2.2 mSv. In light of these calculations, the CBCT acquisition gave a very similar dose to the MSCT acquisition and this is in line with other studies, which concluded that the effective dose of CBCT is lower than or similar to that of MSCT.^17^,^18^

Having technology such as CBCT in a hybrid operating theatre is a useful addition to its capabilities. Open procedures and operations can be performed with direct access to high-quality digital subtraction imaging using the C-arm and flat panel detector. Three-dimensional imaging using the CBCT function, if fitted, is also available if required. In this particular case, it may have been more beneficial to take the patient to the hybrid theatre first. If the closed reduction was unsuccessful, it could easily be converted into an open procedure without moving the patient. High-quality digital subtraction angiography (DSA) of the brachiocephalic artery was also immediately available, should there have been any vascular compromise.

The hybrid operating theatre is a relatively new concept and most of these units have been developed to aid endovascular or cardiovascular procedures.^19^ Almost any percutaneous or closed procedure that requires x-ray imaging could potentially be performed in the hybrid theatre, particularly those that are high risk and may need to be converted into a full open procedure requiring high sterility. A good example is percutaneous aortic valve replacements, which are relatively quick procedures that have good outcomes. Hospital length of stay is as low as a couple of days, in comparison to up to 10 days for an open heart valve replacement. However, potential complications such as femoral artery rupture or valve prosthesis embolization are real and require urgent surgical intervention should they occur. The European Association of Cardio-Thoracic Surgery recommends the use of hybrid theatres for such procedures.^20^ These procedures also routinely use CBCT for alignment and measurements of the native valve immediately prior to implantation.^11^

This novel use of a hybrid theatre with 3D capabilities, situated in a mixed-case theatre complex provided, this patient with an alternative to open reduction surgery or being transferred to the medical imaging department for multi-slice CT. To our knowledge, this is the first reported case of a hybrid theatre and CBCT being utilized in this way for posterior SC joint dislocation.

## Conclusion

There is a growing trend in minimally invasive and percutaneous procedures that require the hybrid operating theatre concept. This case study demonstrates that relatively new additional technologies such as CBCT and the hybrid operating theatre can be utilized for procedures that they were not originally intended for. Their capabilities are only just being fully utilized and they could aid a host of different imaging examinations, not just endovascular and cardiovascular procedures.

## References

[1] Hoekzema N, Torchia M, Adkins M, Cassivi S (2008). Posterior sternoclavicular joint dislocation. Can J Surg.

[2] Rajaratnam S, Kerins M, Apthorp L (2002). Posterior dislocation of the sternoclavicular joint: A case report and review of the clinical anatomy of the region. Clin Anat.

[3] Cope R, Riddervold HO, Shore JL, Sistrom C (1991). Dislocations of the sternoclavicular joint: Anatomic basis, etiologies, and radiologic diagnosis. J Orthop Trauma.

[4] Orth R, Wallace M, Kuo M (2008). C-arm cone beam CT: General principles and technical considerations for use in interventional radiology. J Vasc Interv Radiol.

[5] Maier D, Jaeger M, Izadpanah K, Bornebusch L, Sudkamp N (2011). Traumatic injuries of the sternoclavicular joint. Unfallchirurg.

[6] Groh GI, Wirth MA (2011). Management of traumatic sternoclavicular joint injuries. J Am Acad Orthop Surg.

[7] Feldkamp LA, Davis LC, Kress JW (1984). Practical cone-beam algorithm. J Opt Soc Am.

[8] Ning R, Chen B, Yu R, Conover D, Tang X, Ning Y (2000). Flat panel detector based cone-beam volume CT angiography imaging: System evaluation. IEEE Trans Med Imaging.

[9] Hiwatashi A, Yoshiura T, Noguchi T, Osamu T, Yamashita K, Kamano H, Honda H (2008). Usefulness of cone-beam ct before and after percutaneous vertebroplasty. AJR.

[10] Eide KR, Ødega A, Myhre HO, Haraldseth O (2007). Initial observations of endovascular aneurysm repair using dyna-CT. J Endovasc Ther.

[11] Poon KK, Crowhurst J, James C (2012). Impact of optimising fluoroscopic implant angles on paravalvular regurgitation in transcatheter aortic valve replacements – Utility of three-dimensional rotational angiography. EuroIntervention.

[12] Nordon I, Hinchliffe R, Malkawi A, Taylor J, Holt P, Morgan R, Loftus I, Thompson M (2010). Validation of DynaCT in the morphologic assessment of abdominal aortic aneurysm for endovascular repair. J Endovasc Ther.

[13] Jaffray A, Siewerdsen J, Wong J, Martinez A (2002). Flat-panel cone-beam computed tomography for image guided radiation therapy. Int J Radiat Oncol Biol Phys.

[14] Maki K, Inou N, Takanishi A, Miller AJ (2003). Computer-assisted simulations in orthodontic diagnosis and the application of a new cone beam X-ray computed tomography. Orthod Craniofac Res.

[15] Stratemann SA, Huang JC, Maki K, Miller AJ, Hatcher DC (2008). Comparison of cone beam computed tomography imaging with physical measures. Dentomaxillofac Radiol.

[16] Smyth M, Sutton D, Houston J (2006). Evaluation of the quality of CT-like images obtained using a commercial flat panel detector system. Biomed Imaging Interv J.

[17] Bai M, Liu B, Mu H, Liu X, Jiang Y (2012). The comparison of radiation dose between C-arm flat-detector CT (DynaCT) and multi-slice CT (MSCT): A phantom study. Eur J Radiol.

[18] Wielandts J, De Buck S, Ector J, LaGerche A, Willems R, Bosmans H, Heidbuchel H (2010). Three-dimensional cardiac rotational angiography: Effective radiation dose and image quality implications. Europace.

[19] Kpodonu J (2010). Hybrid cardiovascular suite: The operating room of the future. J Card Surg.

[20] Vahanian A, Alfieri O, Al-Attar N (2008). Transcatheter valve implantation for patients with aortic stenosis: A position statement from the European Association of Cardio-Thoracic Surgery (EACTS) and the European Society of Cardiology (ESC), in collaboration with the European Association of Percutaneous Cardiovascular Interventions (EAPCI). Eur J Cardiothorc Surg.

